# Quercetin inhibits HGF/c-Met signaling and HGF-stimulated melanoma cell migration and invasion

**DOI:** 10.1186/s12943-015-0367-4

**Published:** 2015-05-14

**Authors:** Hui-Hui Cao, Chi-Yan Cheng, Tao Su, Xiu-Qiong Fu, Hui Guo, Ting Li, Anfernee Kai-Wing Tse, Hiu-Yee Kwan, Hua Yu, Zhi-Ling Yu

**Affiliations:** Jockey Club School of Chinese Medicine Building, 7 Baptist University Road, Kowloon Tong, Kowloon, China

**Keywords:** Quercetin, Melanoma, Migration, Invasion, Metastasis, c-Met

## Abstract

**Background:**

Melanoma is notorious for its propensity to metastasize, which makes treatment extremely difficult. Receptor tyrosine kinase c-Met is activated in human melanoma and is involved in melanoma progression and metastasis. Hepatocyte growth factor (HGF)-mediated activation of c-Met signaling has been suggested as a therapeutic target for melanoma metastasis. Quercetin is a dietary flavonoid that exerts anti-metastatic effect in various types of cancer including melanoma. In a previous report, we demonstrated that quercetin inhibited melanoma cell migration and invasion *in vitro*, and prevented melanoma cell lung metastasis *in vivo*. In this study, we sought to determine the involvement of HGF/c-Met signaling in the anti-metastatic action of quercetin in melanoma.

**Methods:**

Transwell chamber assay was conducted to determine the cell migratory and invasive abilities. Western blotting was performed to determine the expression levels and activities of c-Met and its downstream molecules. And immunoblotting was performed in BS^3^ cross-linked cells to examine the homo-dimerization of c-Met. Quantitative real-time PCR analysis was carried out to evaluate the mRNA expression level of HGF. Transient transfection was used to overexpress PAK or FAK in cell models. Student’s *t*-test was used in analyzing differences between two groups.

**Results:**

Quercetin dose-dependently suppressed HGF-stimulated melanoma cell migration and invasion. Further study indicated that quercetin inhibited c-Met phosphorylation, reduced c-Met homo-dimerization and decreased c-Met protein expression. The effect of quercetin on c-Met expression was associated with a reduced expression of fatty acid synthase. In addition, quercetin suppressed the phosphorylation of c-Met downstream molecules including Gab1 (GRB2-associated-binding protein 1), FAK (Focal Adhesion Kinase) and PAK (p21-activated kinases). More importantly, overexpression of FAK or PAK significantly reduced the inhibitory effect of quercetin on the migration of the melanoma cells.

**Conclusions:**

Our findings suggest that suppression of the HGF/c-Met signaling pathway contributes to the anti-metastatic action of quercetin in melanoma.

**Electronic supplementary material:**

The online version of this article (doi:10.1186/s12943-015-0367-4) contains supplementary material, which is available to authorized users.

## Background

The incidence and mortality rates of melanoma have increased world-wide in the last 30 years [1]. Melanoma is notorious for its propensity to metastasize. Early stage melanoma is readily treatable, but advanced metastatic melanoma becomes resistant to treatment. It is reported that the long-term survival rate for patients with metastatic melanoma is only 5% [2]. Currently available chemotherapeutic approaches for melanoma often carry tolerance, low response rate [3] and high toxicity [4,5]. New targeted therapies with high response rate and low toxicity are urgently needed for managing malignant melanoma.

Recently, the role of receptor tyrosine kinase c-Met in melanoma pathogenesis has been gaining interest. c-Met is a cell surface receptor consists of a 50-kDa extracellular α chain and a 140-kDa membrane-spanning β chain, and is synthesized from a single-chain 170-kDa precursor [6]. Binding of HGF (hepatocyte growth factor), the only known endogenous ligand of c-Met [7], to c-Met leads to c-Met homo-dimerization and auto-phosphorylation. The phosphorylated regions of c-Met then act as the multifunction docking site for adaptor molecules which propagate a signaling cascade through a number of effector proteins [8]. Dysregulation of c-Met has been found in many types of cancer, which usually correlated with a poor prognosis [9]. Interestingly, abnormal activation of c-Met signaling is implicated in the acquisition of tumorigenic and metastatic phenotypes in tumors [10,11]. Examinations indicated that c-Met was expressed and activated in melanoma tissues and cell lines [12]. Studies showed that overexpression of c-Met was associated with melanoma growth and metastasis [13,14]. Constitutive activation of c-Met signaling has been reported to promote melanoma metastasis in mice [15,16], while inhibition of c-Met signaling with a specific small molecule tyrosine kinase inhibitor reduced growth and metastasis of experimental human melanoma [17,18]. Blockade of c-Met signaling with the specific small interfering (si) RNA also induced melanoma cell differentiation and prevented melanoma metastasis in a mouse model [17,18]. These studies suggest that c-Met is a therapeutic target for melanoma metastasis.

The dietary flavonoid quercetin (3,3’,4’,5,7-pentahydroxyflavone) is a bioactive compound that wildly distributed in the plant kingdom. It possesses low intrinsic toxicity and does not have carcinogenic activity *in vivo* [19]. Besides, it has a relatively high oral bioavailability [20]. Quercetin has many biological functions including anti-melanoma activity [21]. Several studies showed that quercetin inhibited melanoma growth [22-24] and metastasis [25,26]. Moreover, quercetin also inhibited HGF-induced c-Met phosphorylation in human medulloblastoma cell line DAOY [27], and suppressed HGF-stimulated migration and invasion in DAOY cells [27] and human hepatoma HepG2 cells [28].

Our published data [29] demonstrated that quercetin inhibited melanoma cell migration and invasion *in vitro* and prevented melanoma lung metastasis *in vivo*. Here, we show that quercetin inhibits HGF/c-Met signaling manifested by suppressing c-Met phosphorylation, interfering c-Met dimerization, reducing c-Met protein expression and attenuating the activities of downstream molecules including Gab1, FAK and PAK, which contributes to the anti-metastatic action of quercetin in melanoma.

## Results

### Quercetin suppressed HGF-stimulated melanoma cell migration and invasion

**Figure 1:**
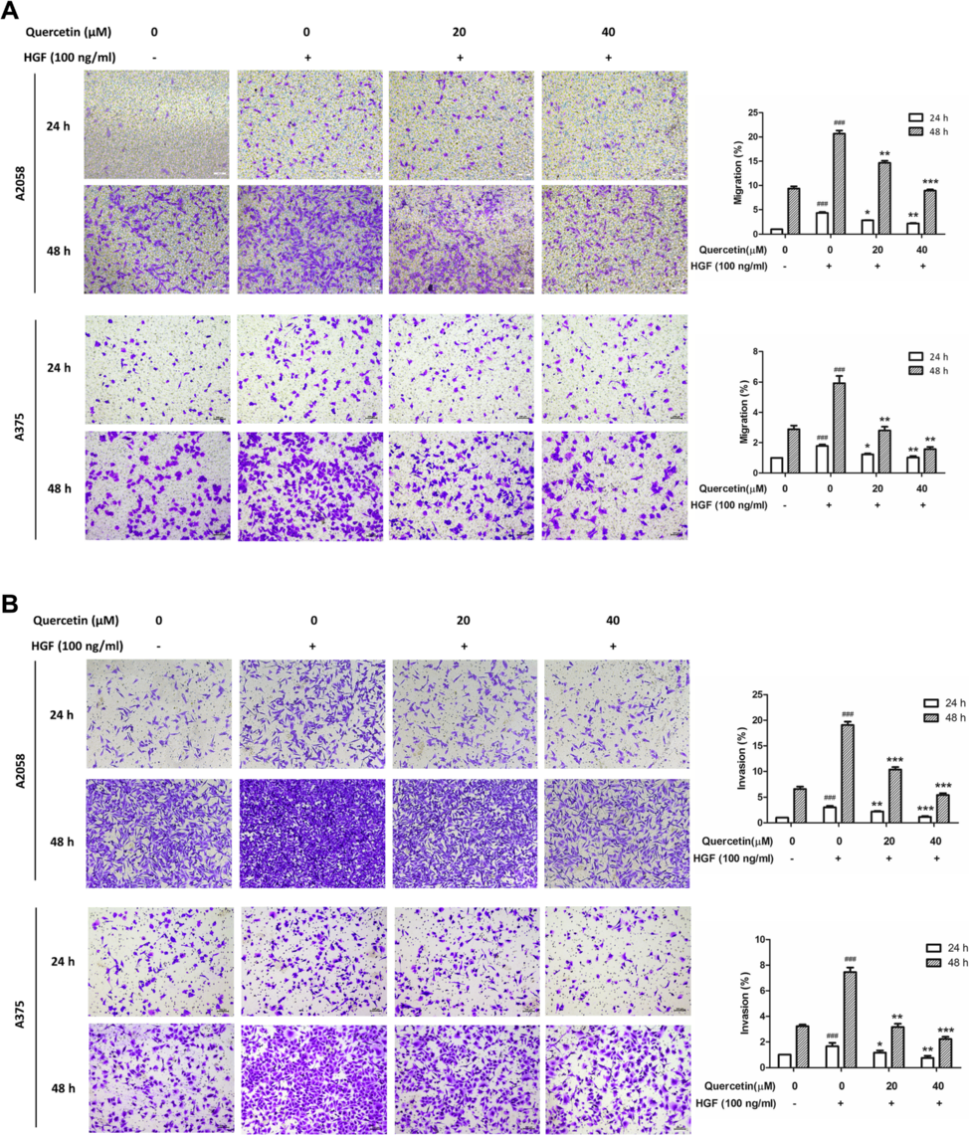
Quercetin suppressed HGF-stimulated melanoma cell migration and invasion. A2058 and A375 cells were seeded onto the upper chamber consisting of 8 μm pore-size filters coated without **(A)** and with **(B)** Matrigel basement membrane matrix, then treated with indicated concentration of quercetin for 24 h or 48 h without (−) or with (+) 100 ng/ml HGF in the lower chamber. The relative quantitative determinations of migrated and invasive cells were calculated with 5 fields counted per experiment. Data were shown as mean ± S.D. from three independent experiments, ^###^ *P* < 0.001 compared with unstimulated and untreated cells, *P < 0.05, **P < 0.01 and ***P < 0.001 compared with stimulated and untreated cells.

The effects of quercetin on HGF-stimulated melanoma cell migration and invasion were determined by the Transwell chamber assays. As shown in Figure 1A, HGF significantly enhanced the migratory abilities in melanoma A2058 and A375 cells. After a 24-h stimulation with HGF, the numbers of A2058 and A375 cells that migrated through the membranes were 4.3-fold and 1.8-fold more than that under unstimulated condition, respectively. A 48-h stimulation with HGF also caused a significant increase in cell migration, whereas treatment with quercetin reduced cell migratory abilities in a dose-dependent manner. In parallel, a Matrigel invasion assay showed that stimulation with HGF significantly increased the invasiveness of melanoma cells at both 24 h- and 48 h-incubation periods, and this effect was dose-dependently reverted by quercetin treatments (Figure 1B). Under all these conditions, quercetin did not affect cell proliferation (Additional file 1: Figure S1). These results indicate that quercetin dose-dependently inhibits HGF-stimulated melanoma cell migration and invasion.

### Quercetin reduced c-Met phosphorylation and dimerization

**Figure 2:**
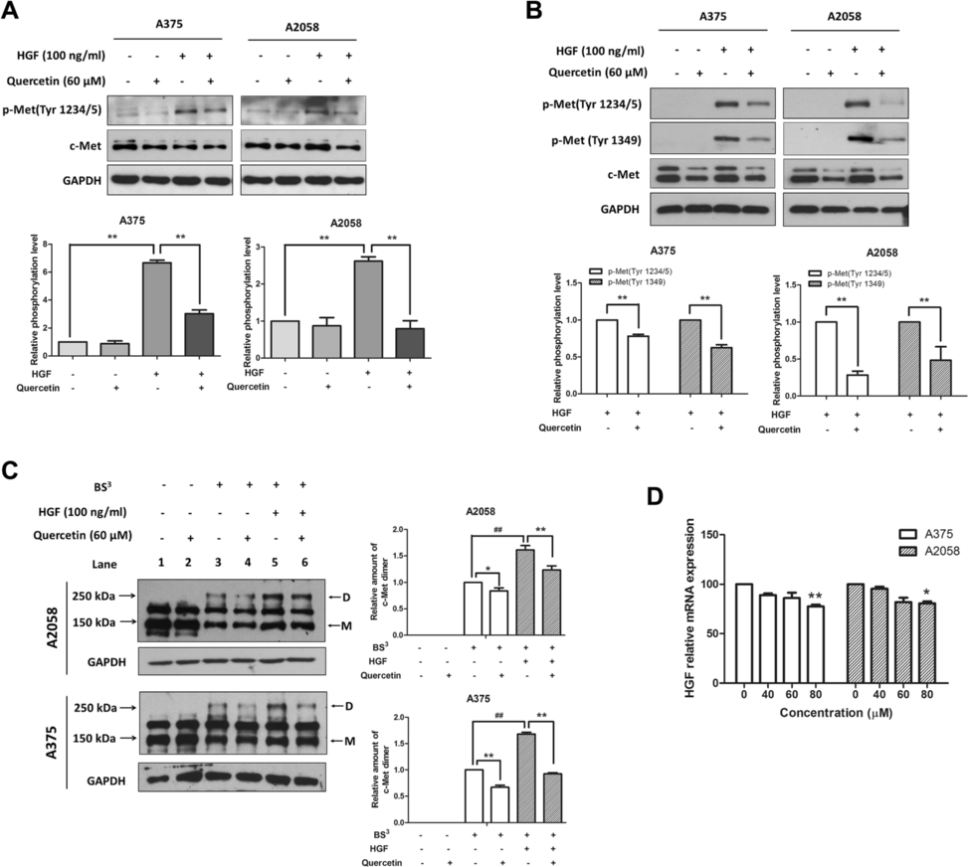
Quercetin inhibited the activation of c-Met in melanoma cells. **(A)** Cells were treated with the indicated concentrations of quercetin for 6 h and then stimulated with or without HGF (100 ng/ml) for 10 min. Levels of c-Met and phospho-Met in total lysates were evaluated by immunoblotting. The representative results (upper) and the relative phosphorylation levels (bottom) were shown. The relative levels of phospho-Met were calculated after normalizing the levels of phospho-Met to total c-Met. **(B)** Cells were grown in serum-free medium overnight before incubation with the indicated concentrations of quercetin for 6 h, after stimulation with or without 100 ng/ml HGF for 10 min, cells were lysed for immunoblotting. The representative results (upper) and the relative phosphorylation levels (bottom) were shown. The relative levels of phospho-Met were calculated after normalizing the levels of phospho-Met to the total c-Met. **(C)** Quercetin reduced c-Met homo-dimerization. Cells were starved overnight and then treated with indicated concentrations of quercetin for 6 h. After that, these cells were stimulated with or without HGF (100 ng/ml) for 10 min, and then incubated with BS^3^. Cell lysates were prepared for immunoblotting by using an anti-c-Met antibody. The arrows indicated the dimer **(D)** and the monomer (M) c-Met. Molecular weights of the marker are shown on the left. The representative results (left) and the relative expression levels of dimer c-Met (right) were shown. The relative levels of dimer c-Met were analyzed after normalizing the levels of dimer c-Met to monomer c-Met. **(D)** Effect of quercetin on HGF mRNA expression. Cells were treated with indicated concentrations of quercetin for 24 h and then the real-time PCR analysis was performed to detect the mRNA expression levels of HGF. Data were mean ± S.D. from three independent experiments. *P < 0.05, **P < 0.01 and ^##^ *P* < 0.01.

It has been reported that activation of the HGF/c-Met pathway promotes cell invasion, migration and allows cancer metastasis [9]. Therefore, we examined if quercetin inhibited the HGF/c-Met signaling pathway. Cells were treated with either the vehicle or quercetin (60 μM) for 6 h, and then stimulated with or without HGF (100 ng/ml) for 10 min. Figure 2A showed that unstimulated cells had low c-Met phosphorylation level at the major auto-phosphorylation sites Tyr1234/5, while stimulation with HGF significantly increased c-Met phosphorylation. Interestingly, pre-treatment with quercetin reduced the phosphorylation of c-Met in the HGF-stimulated cells. To eliminate the influence of serum growth factors, cells were grown in serum-free medium overnight and then treated with either the vehicle or quercetin (60 μM) for 6 h before stimulating with 100 ng/ml HGF for 10 min. Data from the Western blot analysis showed that c-Met hardly phosphorylated in starved cells and stimulation with HGF remarkably increased c-Met phosphorylation levels at the Tyr1234/5 sites and the multi-substrate docking site Tyr1349, whereas pre-incubation with quercetin decreased the phosphorylation levels of c-Met in the HGF-stimulated cells (Figure 2B). In addition, we observed that total c-Met expression levels were reduced by quercetin treatment (Figure 2A and B).

Next, we determined if quercetin affected c-Met dimerization. As shown in Figure 2C, incubation with a cross linker BS^3^ increased c-Met dimerization (lane 3 versus 1), while pretreatment with quercetin reduced the expression level of c-Met dimer (lane 4 versus 3). Besides, stimulation with HGF caused an apparent c-Met dimerization (lane 5 versus 3), which was also inhibited by quercetin pre-incubation (lane 6 versus 5).

It is reported that most of melanoma cells produce HGF that induces sustained activation of its receptor c-Met [30]. We wondered if quercetin affected the endogenous HGF expression level. Real-time PCR data showed that the mRNA expression levels of HGF in melanoma A375 and A2058 cells were slightly reduced after treating with quercetin for 24 h (Figure 2D).

Taken together, these findings suggest that quercetin inhibits c-Met activation probably mainly by inhibiting c-Met phosphorylation and dimerization.

### The inhibitory effect of quercetin on c-Met expression was associated with reduced expression of fatty acid synthase

**Figure 3:**
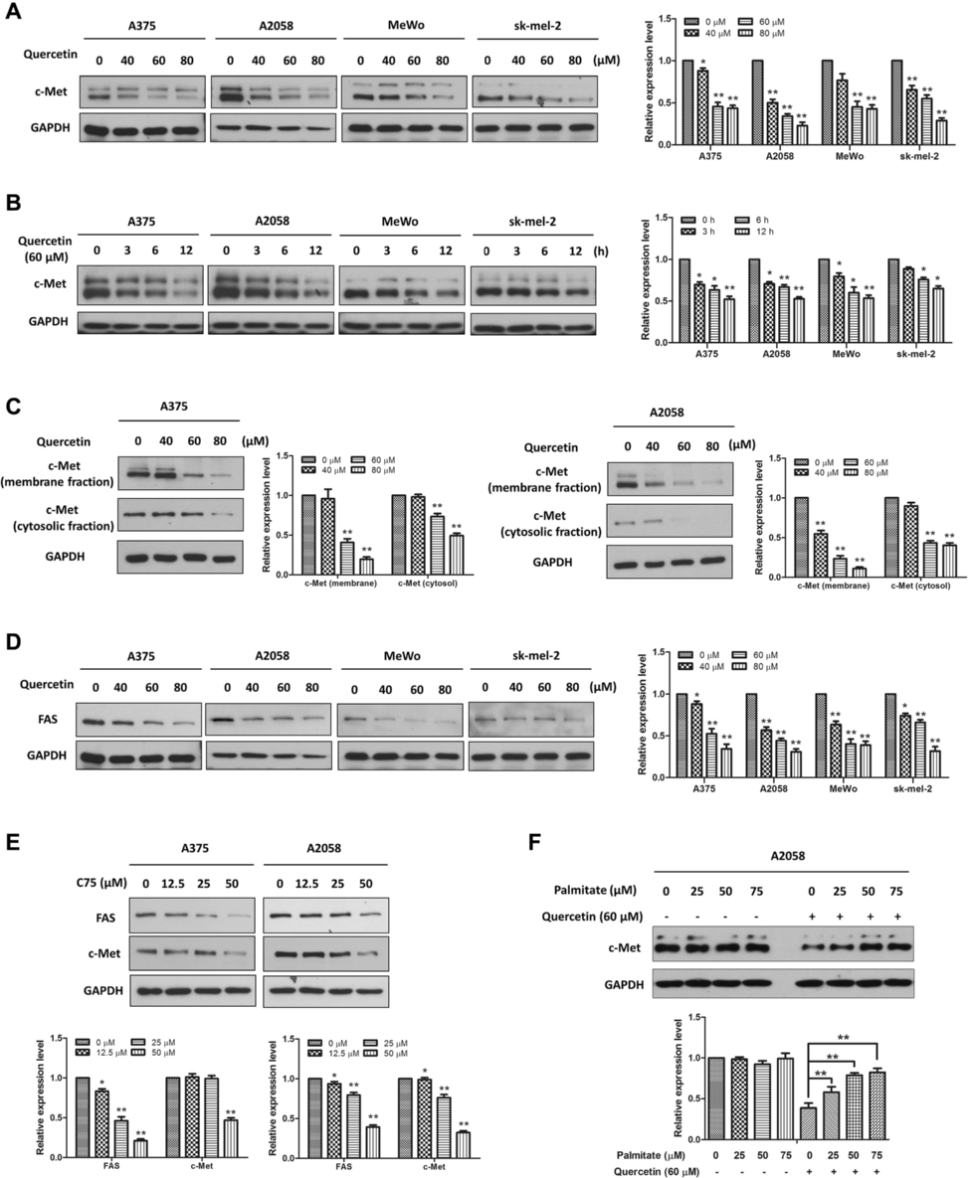
Quercetin reduced c-Met levels through the inhibition of FAS. A375, A2058, MeWo and sk-mel-2 cells were treated with **(A)** various concentrations of quercetin for 24 h or **(B)** a fixed concentration (60 μM) for various durations, and then the whole-cell lysates were prepared and the Western blot analysis was performed to determine the expression level of c-Met. **(C)** A375 and A2058 cells were treated with indicated concentrations of quercetin for 24 h, and then the membrane and cytosolic lysates were prepared. The expression levels of c-Met in the two fractions were examined by immunoblotting. **(D)** A375, A2058, MeWo and sk-mel-2 cells were treated with indicated concentrations of quercetin for 24 h, and then the expression of FAS were determined by immunoblotting. **(E)** A375 and A2058 cells were treated with indicated concentrations of C75 for 24 h and then the immunoblotting assay was conducted to determine the expression levels of FAS and c-Met. **(F)** A2058 cells were exposed to either the vehicle or 60 μM quercetin for 24 h in the absence or presence of palmitate, and then the whole-cell lysates were prepared for immunoblotting by using a c-Met antibody. Independent experiments were performed at least three times, and the results from a representative experiment are shown. The relative expression levels were analyzed by Image J software and shown as mean ± S.D., *P < 0.05, **P < 0.01.

To verify the effect of quercetin on c-Met expression, we performed the Western blot analysis in melanoma MeWo and sk-mel-2 cells in addition to A375 and A2058 cells. Whole-cell lysates from the four cells treated with various concentrations of quercetin (0, 40, 60 and 80 μM) for 24 h, or a fixed concentration (60 μM) for various durations (0, 3, 6 and 12 h) were immuno-blotted with the c-Met antibody. We found that quercetin reduced c-Met expression in both dose- and time-dependent manners in these four cell lines (Figure 3A and B). Since c-Met is a membrane receptor tyrosine kinase, we examined if quercetin inhibited cell surface c-Met expression. After treating A375 and A2058 cells with the indicated concentrations of quercetin for 24 h, we isolated the membrane and cytosolic fractions for Western blot analyses. As shown in Figure 3C, the expression levels of c-Met were dose-dependently reduced by quercetin in both membrane and cytosolic fractions of the A375 and A2058 cells.

It has been reported that palmitate whose synthesis requires fatty acid synthase (FAS) involvement is essential for maintaining c-Met expression, and inhibition of FAS using inhibitors or the shRNA knockdown greatly reduces c-Met expression [31]. The activity of FAS is tightly correlated with melanoma progression and metastasis. Studies showed that malignant melanomas expressed higher levels of FAS than nevi, and metastatic melanomas expressed the highest levels of FAS [32]. Moreover, increased FAS expression was usually associated with melanoma invasion depth and poor patient survival [32-34]. Quercetin has been reported to inhibit FAS activity, which was associated with quercetin-mediated prostate cancer cell apoptosis [35]. Quercetin also reduced FAS expression levels and inhibited cell proliferation in nasopharyngeal carcinoma cells [36]. Interestingly, in this study, quercetin not only down-regulated c-Met expression levels, but also reduced FAS expression in human melanoma A375, A2058, MeWo and sk-mel-2 cells (Figure 3D). We suggested that quercetin-afforded down-regulation of c-Met was probably caused by inhibiting FAS. As expected, C75, a specific inhibitor of FAS, inhibited c-Met with kinetics similar to that for inhibiting FAS (Figure 3E). More importantly, addition of palmitate reduced quercetin-mediated c-Met reduction in a dose-dependent manner (Figure 3F). These results suggest that inhibition of FAS expression contributes to the inhibitory effect of quercetin on c-Met expression.

### Quercetin suppressed the activation of c-Met downstream molecules

**Figure 4:**
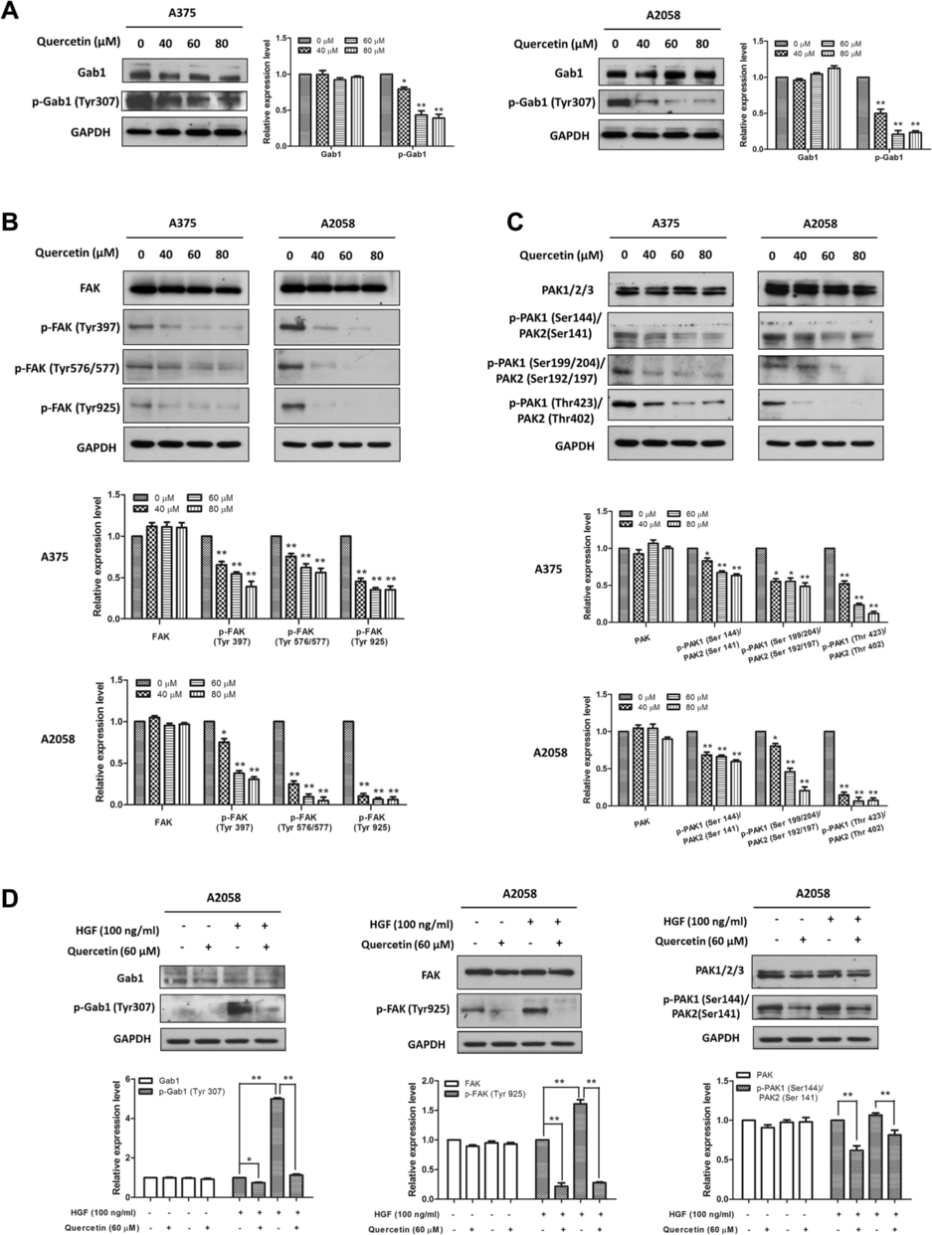
Quercetin suppressed the activation of c-Met downstream molecules. (**A**, **B** and **C**) A375 and A2058 cells were treated with indicated concentrations of quercetin for 24 h. **(D)** A2058 cells were starved overnight and then exposed to the vehicle or quercetin (60 μM) for 6 h. After that, these cells were stimulated with or without HGF (100 ng/ml) for 10 min. For each experiment, the whole-cell lysates were prepared and probed by Western blot using specific antibodies. Independent experiments were performed at least three times, and the results from a representative experiment are shown. The relative expression levels were analyzed by Image J software and shown as mean ± S.D., *P < 0.05, **P < 0.01.

After phosphorylation, c-Met recruits adaptor protein Gab1 (Grb2-associated binding protein 1), phosphorylates Gab1 at tyrosine site 307 and hence activates downstream FAK (Focal Adhesion Kinase) and PAK (p21-activated kinase). Activation of FAK and PAK pathways results in increased cell motility, migration and invasion [9]. To determine if quercetin inhibited the c-Met downstream molecules, A375 and A2058 cells were treated with various concentrations of quercetin for 24 h and the whole-cell lysates were prepared for Western blot analyses. We found that the levels of phosphorylated Gab1 at the tyrosine 307 (Tyr307) site in both A375 and A2058 cells were reduced in a dose-dependent manner (Figure 4A), and the activation of both FAK and PAK were inhibited by quercetin treatment in a dose-dependent manner (Figure 4B and C). To eliminate the influence of serum growth factors, cells were starved overnight and treated with either the vehicle or quercetin (60 μM) in serum-free medium for 6 h, followed by stimulation with HGF (100 ng/ml) for 10 min. Western blot analyses showed that under the starved conditions, the phosphorylation of Gab1 was significantly reduced in A2058 cells, while stimulation with HGF resulted in a remarkable increase in Gab1 phosphorylation which was completely abolished by quercetin pre-treatment (Figure 4D, left). The activation of FAK and PAK in A2058 cells were significantly inhibited by quercetin under both HGF-stimulated and unstimulated conditions (Figure 4D, middle and right). These data demonstrate that quercetin suppresses the activation of c-Met downstream molecules Gab1, FAK and PAK.

### The inhibitory effect of quercetin on cell migration was partially reversed by PAK or FAK overexpression

**Figure 5:**
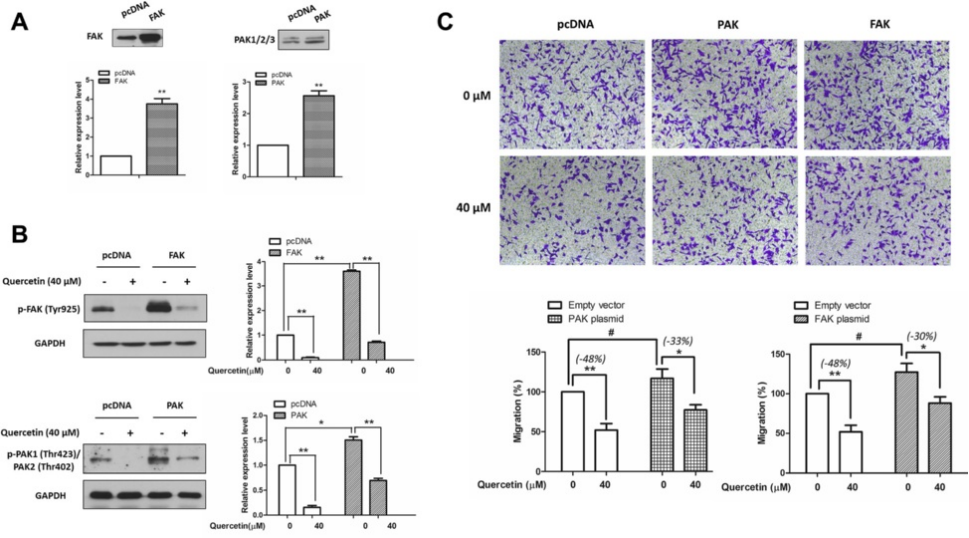
Overexpression of PAK or FAK partially reversed the migration-inhibition effect of quercetin. A2058 cells were transiently transfected with a FAK-expressing construct, a PAK-expressing construct, or an empty vector for 24 h. **(A)** Expression levels of FAK (or PAK) on cells that transfected with FAK (or PAK)-expressing construct. **(B)** After transfection, cells were treated with quercetin for 24 h, and the effects of quercetin on FAK (or PAK) overexpressed cells were determined by immunoblotting. **(C)** Cell migratory abilities were measured by migration chamber assay after treated with quercetin for 48 h. The relative quantitative determinations of migrated cells were calculated with 5 fields counted per experiment. The data shown here are the mean ± S.D. from three independent experiments. ^#^ *P* < 0.05, *P < 0.05 and ** < 0.01.

To better understand the involvement of c-Met signaling in quercetin-mediated anti-metastatic effects, we investigated whether overexpression of PAK or FAK reversed the quercetin-mediated inhibitory effect on migration. A2058 cells were transiently transfected with either a FAK-expressing construct (or a PAK-expressing construct) or an empty vector. After 24 h transfection, the expression of FAK and phospho-FAK were increased remarkably, and the quercetin-mediated FAK inhibition was also partially reversed (Figure 5A and B). Similar results were observed in the PAK-expressing construct transfected cells, overexpression of PAK reversed quercetin-induced FAK inhibition (Figure 5A and B). Furthermore, cells that transfected with PAK or FAK constructs showed a slight but significant increase in the migratory abilities as compared with cells that were transfected with empty vector (^#^*P* < 0.05). Quercetin treatment (40 μM) inhibited the cell migratory abilities. This inhibitory effect was reduced from 48% to 33% in PAK overexpressing cells, and from 48% to 30% in FAK overexpressing cells. These data indicate that the inhibitory effect of quercetin on migration is partially reduced by the overexpression of PAK or FAK in melanoma cells.

## Discussion

The HGF/c-Met pathway is activated in various types of cancer, which stimulates cancer cell growth and metastasis [9]. HGF is a multifunctional cytokine acting as a mitogen, motogen and morphogen [37]. Most cancers express both HGF and c-Met, leading to autocrine activation of c-Met. Besides, aberrant c-Met activation can also be achieved through c-Met overexpression, activating c-Met mutations, or c-Met gene amplification [38]. In melanoma, HGF and c-Met are expressed [9] and involved in tumorigenesis [30]. In this study, we found that quercetin, a widely existed dietary flavonoid, suppressed c-Met signaling by inhibiting c-Met phosphorylation and dimerization (Figure 2A, B and C). Quercetin also inhibited HGF-stimulated melanoma cell migration and invasion (Figure 1), which was in agreement with the previous studies that quercetin inhibited HGF-stimulated migration and invasion in human medulloblastoma cell DAOY [27] and human hepatoma HepG2 cells [28]. In addition, many other known flavonoids, such as EGCC [39], luteolin [28,31], kaempferol [27] and myricetin [27] also showed inhibitory effects on HGF-stimulated cancer cells migration. These observations indicated that these plant-derived flavonoids shared similar activities and may be useful in melanoma treatment and prevention.

Since some melanoma cells were reported to express HGF and secret a detectable level of HGF to induce constitutive activation of c-Met [30], we wondered if quercetin exerted its effects by affecting HGF autocrine. We collected the culture medium of quercetin-treated A375 and A2058 cells, and examined the HGF levels by ELISA, but the level of secreted HGF was too low to be detected. Besides, we also found that the inhibitory effect of quercetin on HGF mRNA expression was not obvious (Figure 2D). Based on these results we could not draw a conclusion regarding the impact of HGF autocrine on quercetin-mediated c-Met signaling inhibition.

We found that c-Met protein levels were decreased after quercetin treatment in both dose- and time-dependent manners (Figure 3A and B). Since melanoma can be divided into three mutually exclusive genetic subsets: BRAF mutant melanoma, NRAS mutant melanoma and melanoma of wild type at both loci [40], to confirm the generality of this finding, beside two BRAF mutant melanoma cell lines A375 and A2058, NRAS mutant melanoma cell line sk-mel-2 and wild type NRAS and BRAF melanoma cell line MeWo with constant c-Met activation [12] were also used. Results showed that treatment with quercetin down-regulated the expression levels of c-Met in all these four cell lines (Figure 3A and B), suggesting that the inhibitory effect of quercetin on c-Met receptor is a general phenomenon in melanoma. It was further found that c-Met expression was higher in membrane fractions than in cytosol fractions, and c-Met in both fractions were inhibited by quercetin treatment (Figure 3C). We also found that quercetin did not affect the mRNA expression levels of c-Met in all these cell lines (data not shown), which indicated that quercetin post-transcriptionally down-regulated c-Met expression. Coleman *et al.* identified a regulatory link between FAS and c-Met. They found that inhibition of FAS by using inhibitors (luteolin or C75) or the shRNA knockdown approach can down-regulate c-Met expression in human prostate cancer cells, and the production of the 16-carbon fatty acid palmitate by FAS is required for maintaining c-Met expression [31]. Similar results have also been observed in diffuse large B cell lymphoma by Uddin *et al.* [41] and in breast cancer by Hung *et al.* [42]. Furthermore, Coleman *et al.* found that all the flavonoids luteolin, apigenin, and quercetin, which possess a same moiety with a C2-C3 double bond in the C-ring, reduced c-Met expression in human prostate cancer cells [31]. In this study, we found that quercetin reduced c-Met expression, C75, a specific inhibitor of FAS, showed similar inhibitory effect on the expression of FAS and c-Met (Figure 3E), and exogenous palmitate prevented quercetin-induced reduction of c-Met (Figure 3F), further supporting a role of FAS in maintaining c-Met expression levels. However, the mechanism by which FAS inhibition decreases c-Met expression is not yet clear. A possible explanation is that FAS inhibition may cause an imbalance in the membrane phospholipids levels, which may result in decreased c-Met membrane localization [41,43]. Lipid rafts are membrane microdomains that serve as platforms for cell signaling, and FAS was shown to regulate the activity of lipid rafts [44]. Recent studies found that altering the structure or function of lipid rafts prevented the activation of c-Met [45]. Quercetin is also reported to suppress lipid biosynthesis in breast cancer MDA-MB-231 cells [35]. Therefore, the quercetin-mediated reduction of c-Met in melanoma cells may be due to FAS inhibition.

After phosphorylation on tyrosine site 1349, c-Met becomes a docking site for recruiting Gab1, which further activates downstream FAK and PAK [9]. Activation of both c-Met/Gab1/FAK and c-Met/Gab1/PAK signalings promotes tumor metastasis [9]. Our data showed that quercetin dose-dependently decreased the levels of phospho-Gab1, phospho-FAK and phospho-PAK (Figure 4A, B and C), suggesting that inhibition of the c-Met/Gab1/FAK and c-Met/Gab1/PAK pathways may contribute to the anti-metastatic effects of quercetin. It is well-known that quercetin has multiple targets including receptor tyrosine kinases, matrix metalloproteinase, mitochondria and other signaling enzymes [46]. Besides Gab1, c-Met can also activate other molecules such as STAT3 [8] which is involved in melanoma metastasis. STAT3 can be suppressed by quercetin treatment as shown in our previous study [29]. Therefore, we could not exclude the possibilities that quercetin inhibits melanoma metastasis by modulating other pathways downstream of c-Met. Indeed, overexpression of FAK or PAK only partially reversed quercetin-mediated inhibitory effects on melanoma cell migration (Figure 5C). Whether overexpression of both PAK and FAK can completely reverse the migration inhibitory effect of quercetin in melanoma cells needs to be further studied.

## Conclusions

In summary, our previous [29] and current studies show that quercetin suppresses melanoma cell migration and invasion. This effect is, at least in part, due to the inhibition of HGF/c-Met signaling. Our findings provide novel insights into the anti-melanoma molecular mechanisms of quercetin, and further suggest a potential role of quercetin in melanoma management.

## Methods

### Reagents and antibodies

Antibodies against phospho-Met (Tyr1234/Y1235), phospho-Met (Tyr1349), phospho-Met (Tyr1003), c-Met, phospho-Gab1 (Tyr307), FAK, phospho-FAK (Tyr576/577), phospho-FAK (Tyr925), phospho-FAK (Tyr397), PAK1/2/3, phospho-PAK1 (Ser144)/PAK2 (Ser141), phospho-PAK1 (Ser199/204)/PAK2 (Ser192/197), phospho-PAK1 (Thr423)/PAK2 (Thr402) and FAS were obtained from Cell Signaling Technology (Beverly, MA, USA). Anti-GAPDH was purchased from Santa Cruz Biotechnology (Santa Cruz, CA, USA). Goat anti-rabbit IgG, goat anti-mouse IgG and protein markers were supplied by Bio-Rad (Hercules, CA, USA). Recombinant human HGF was obtained from PeproTech (PeproTech, NJ, USA). Other chemicals were obtained from Sigma–Aldrich (St. Louis, MO, USA). Quercetin was obtained from Chromadex (USA). The stock solution of 100 mM quercetin was prepared in dimethyl sulfoxide (DMSO) and stored at −20°C. Palmitate was complexed to bovine serum albumin as previously described [47]. In short, sodium palmitate was dissolved in ethanol:H_2_O (1:1, v/v) at 70°C at a final concentration of 150 mM, then the solutions were complexed with fatty-acid-free BSA (10% solution in H_2_O) by stirring for 1 h at 37°C and then diluted in culture medium. The final molar ratio of fatty acid:BSA was 5:1.

### Cell culture

A375, A2058, sk-mel-2 and MeWo cell lines were obtained from the American Type Culture Collection (ATCC, USA), and were incubated in high glucose Dulbecco’s modified Eagle’s medium (DMEM, GIBCO, USA), supplemented with 10% (v/v) fetal bovine serum (FBS, GIBCO, USA) and 1% penicillin/streptomycin (P/S, GIBCO, USA) at 37°C in a humidified atmosphere of 5% CO_2_.

### Cell migration and invasion assay

The cell migratory ability was tested using a commercial Transwell insert (8 μm pore size, Corning, NY, USA). A2058 and A375 cells were suspended in serum-free DMEM medium containing 0.1% BSA. Then 0.1 ml of the cells suspension was added to the top of the Transwell inserts, and 0.5 ml of serum-containing medium with (+) or without (−) HGF (100 ng/ml) was plated in the bottom wells. Quercetin was added to both inserts and wells. The chambers were then assembled and incubated for 24 h or 48 h at 37°C in a 5% CO_2_ incubator. After that, non-invading cells were removed from the upper surface of the membrane by scrubbing. The migrated cells on the underside of the filter were first fixed with 100% methanol and then stained by 0.1% crystal violet solution and counted in five random fields. The relative migration was calculated from the ratio of the migrated cells that quercetin treated versus the vehicle control cells.

For the invasion assay, BD BioCoat™Matrigel™ invasion chamber (24 well plate, 8-μm pore size, BD Biosciences, San Jose, CA, USA) were used. 0.5 ml warm (37°C) serum-free medium was added to both the inserts and the wells to allow the chamber rehydrated at 37°C in a 5% CO_2_ incubator for 2 h. Then A2058 and A375 cells in 0.5 ml serum-free medium containing 0.1% BSA were added to the inserts, while 0.75 ml of serum-containing medium with or without HGF (100 ng/ml) was placed in the lower chambers. Quercetin was added to both the inserts and the lower chambers. Chambers were then assembled and incubated for 24 h or 48 h at 37°C. Subsequent steps were performed in the same manner as described for cell migration assay.

### Western blot analysis

Membrane protein was extracted using Mem-PER™ Plus kit (Thermo Scientific, Rockford, IL, USA) according to the manufacturer’s protocol. Preparation of total protein lysates and Western blot analysis were performed as described previously [29]. Protein concentrations were determined according to the Bio-Rad protein assay reagent. The cell lysates were separated on 6% or 8% gels and transferred to nitrocellulose membranes. The membranes were incubated in 5% skim milk in TBS-T buffer at room temperature. Blocked membranes were incubated with primary antibodies at 4°C overnight, followed by incubation with secondary antibodies at room temperature for 1 hour. After washing in TBS-T, immune-reactive bands were visualized by chemiluminescence substrate (Thermo Scientific, Rockford, IL, USA).

### Real-time PCR

Total RNA was extracted with Trizol reagent (Invitrogen, USA), and reverse-transcripted with oligo-dT using M-MLV reverse transcriptase (Promega, USA) according to the manufacturer’s protocol. Quantitative real time PCR was carried out by monitoring the increase in fluorescence of SYBR green with the ViiA 7 Real Time PCR System (Applied Biosystems, USA). The primer sets were synthesized by Invitrogen, HGF primers: forward TCCCCATCGCCATCCCC and reverse CACCATGGCCTCGGCTGG, GAPDH primers: forward CTGCACCACCAACTGCTTAGC and reverse CTTCACCACCTTCTTGATGTC. Each sample was amplified in triplicate for quantification. Data were analyzed by relative quantitation using the ΔΔC_t_ method and normalized to GAPDH.

### Dimerization of c-Met

The dimerization of c-Met was analyzed as described previously [48]. Melanoma cells were starved overnight and then treated with vehicle control or quercetin for 6 h, followed by stimulation with HGF (100 ng/ml) on ice for 10 min. Subsequently, the cross-linker Bis[sulfosuccinimidyl] substrate (BS^3^, 0.25 mM, Thermo Scientific, Rockford, IL, USA) was added to cells and reacted at 37°C for 5 min. Cells were then transferred on ice for 10 min. After that, non-reactive BS^3^ were quenched with 50 mM Tris–HCl (pH 7.4). Cell lysates were separated by 6% SDS-PAGE and immunoblotted with an anti-c-Met antibody.

### Plasmid transient transfection

Plasmids pCMV6M-Pak1 (Addgene plasmid 12209) was provided by Sells *et al.* [49] and myc-Rapr-FAK (Addgene plasmid 25926) was supplied by Karginov *et al.* [50]. To overexpress FAK and PAK, cells were transfected with plasmids using Lipofectamine 2000 (Invitrogen, USA) according to the manufacturer’s protocol. Empty pcDNA3.0 plasmid was used as mock transfectant. Cells were transfected with plasmids for 24 h or 48 h before functional assays were carried out.

### Statistical analysis

The Student’s *t*-test was used to analyze differences between two groups. All data were presented as means ± S.D. from at least three independent experiments. *P* < 0.05 was considered as statistically significant.

## Additional file

Additional file 1: Figure S1. Effect of quercetin on melanoma cell proliferation. A375 and A2058 cells were treated with indicated concentrations of quercetin for 24 h or 48 h. Cell viability was measured by the MTT assay. Data were mean ± S.D. from three independent experiments.

## Abbreviations

BS^3^: Bis[sulfosuccinimidyl] suberate
EGCG: (−)-epigallocatechin-3-gallate
ELISA: Enzyme-linked immunosorbent assay
FAK: Focal adhesion kinase
FAS: Fatty acid synthase
Gab1: Grb2-associated binding protein 1
HGF: Hepatocyte growth factor
PAK: p21-activated kinase
