# Supplementary figures and images for: Quercetin inhibits HGF/c-Met signaling and HGF-stimulated melanoma cell migration and invasion
Source: Mol Cancer. 2015 May 14;14:103. doi: 10.1186/s12943-015-0367-4 (PMC4435529; doi:10.1186/s12943-015-0367-4)

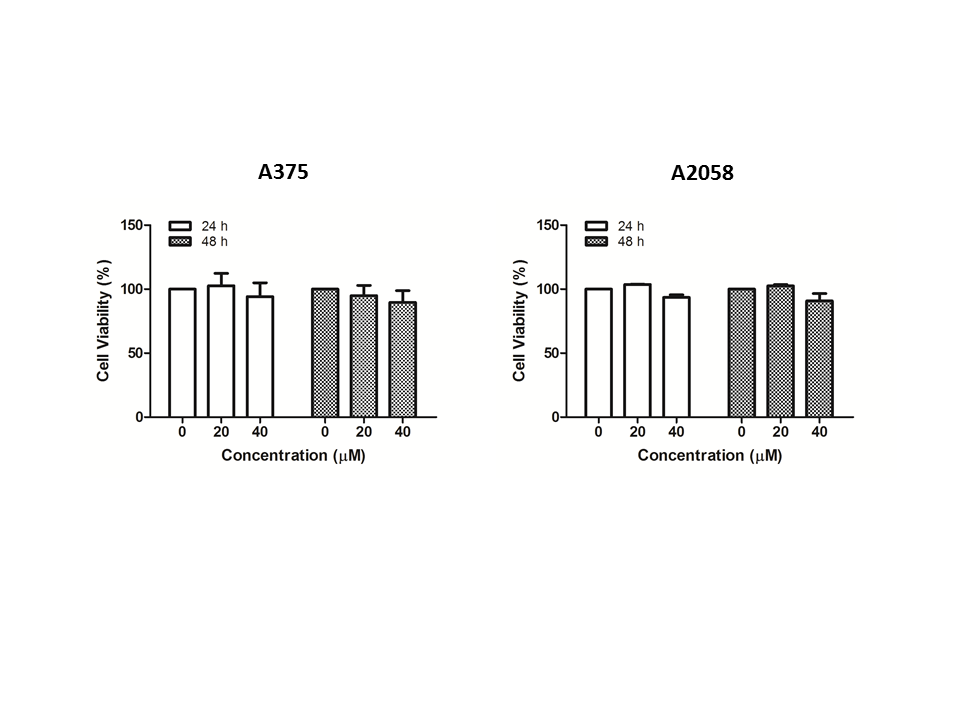

Supplement: Additional file 1: Figure S1. — Effect of quercetin on melanoma cell proliferation. A375 and A2058 cells were treated with indicated concentrations of quercetin for 24 h or 48 h. Cell viability was measured by the MTT assay. Data were mean ± S.D. from three independent experiments. [file 12943_2015_367_MOESM1_ESM.tiff]
